# Tethering of Telomeres to the Nuclear Envelope Is Mediated by SUN1-MAJIN and Possibly Promoted by SPDYA-CDK2 During Meiosis

**DOI:** 10.3389/fcell.2020.00845

**Published:** 2020-09-04

**Authors:** Guishuan Wang, Xiaolong Wu, Liwei Zhou, Sheng Gao, Damin Yun, Ajuan Liang, Fei Sun

**Affiliations:** ^1^Medical School, Institute of Reproductive Medicine, Nantong University, Nantong, China; ^2^Reproductive Medical Center, Tongji Hospital, Tongji University School of Medicine, Shanghai, China

**Keywords:** meiosis, telomere, SUN1, MAJIN, TERB1, CDK2, SPDYA

## Abstract

During meiosis, telomeres attach to the nuclear envelope (NE) to promote homologous chromosome moving, pairing, synapsis, and recombination. The telomere-NE attachment is mediated by SUN1, TERB1-TERB2-MAJIN (TTM complex), and TRF1. The interaction of the TTM complex with shelterin is mediated by TERB1 and TRF1, but how SUN1 interacts with the TTM complex is not yet fully understood. In this study, we found that SUN1 not only interacted with TERB1 but also interacted with MAJIN, and the interaction of SUN1 with MAJIN is stronger than TERB1. We also found that SUN1 interacted with SPDYA, an activator of CDK2. The binding sites of MAJIN and SPDYA at SUN1 were mapped, and both MAJIN and SPDYA bound to the N-terminal domain of SUN1 and the two binding sites were close to each other. Furthermore, SPDYA bound to SUN1 via the Ringo domain and recruited CDK2 to SUN1. Then, we found that the interaction of SUN1 with MAJIN was decreased by the CDK2 inhibitors. Taken together, our results provide the possible mechanism of SUN1, MAJIN, and SPDYA-CDK2 in promoting the telomere-NE attachment during meiosis.

## Introduction

During meiosis, telomeres attach to the nuclear envelope (NE) and cluster to form a bouquet that is conserved and has been found in many organisms (Zickler and Kleckner, 2016). Formation of the bouquet is required for meiosis by tethering telomeres together and facilitating homologous chromosome pairing, synapsis, and recombination (Harper et al., 2004). There are three protein complexes involved in bouquet formation. The LINC (linker of nucleoskeleton and cytoskeleton) complex, which is composed by trans-membrane proteins containing KASH and SUN domains, and localizes at the NE, links the NE to the cytoskeleton where forces are generated to move chromosomes. Moreover, SUN domain proteins provide telomere-anchoring sites at the NE (Hiraoka and Dernburg, 2009; Tapley and Starr, 2013). The shelterin complex localized at the telomeres is required for bouquet formation, especially the core subunit of the shelterin complex, TRF1 (Wang et al., 2018). There is a “linker” bridging LINC and shelterin complexes together. In mouse, this liner consists of TERB1, TERB2, and MAJIN (Conrad et al., 2008; Hiraoka and Dernburg, 2009; Daniel et al., 2014; Shibuya et al., 2015; Wang et al., 2019). Many efforts have been made to reveal the connection between the linker and shelterin complex, indicating that TERB1 and TRF1 contribute to this connection (Shibuya et al., 2014; Long et al., 2017; Pendlebury et al., 2017; Zhang et al., 2017; Dunce et al., 2018). However, the connection between the linker and LINC complex is not yet fully understood (Shibuya et al., 2014).

CDK2 and its activators, SPDYA and Cyclin E, are required for the bouquet formation (Palmer et al., 2019). In the *Cdk2*, *Spdya*, and *Cyclin E* knockout mice, telomeres dissociate from the NE, indicating that the LINC-linker-shelterin connection is broken down, but where the disconnection occurs is unknown (Viera et al., 2015; Manterola et al., 2016; Tu et al., 2017). CDK2 is a cyclin-dependent kinase, and the typical cyclins, including Cyclin E and A, and atypical cyclin, SPDYA, activate the kinase activity of CDK2 (Link et al., 2014; Tu et al., 2017). The cyclins bind to CDK2 and lead to phosphorylation of T160 on the T-loop to activate CDK2, whereas SPDYA binds to and activates CDK2 regardless of the T loop phosphorylation (Gonzalez and Nebreda, 2020). It has been reported that CDK2 phosphorylates SUN1 (Viera et al., 2015; Mikolcevic et al., 2016), implying that CDK2 may function in the LINC-linker connection.

In this research, we determined how LINC and linker complexes connect together. We found that SUN1 not only interacted with TERB1 but also interacted with MAJIN, and the interaction of SUN1 with MAJIN was stronger than TERB1. We also found that SPDYA interacted with SUN1 via the Ringo domain and recruited CDK2 to SUN1. Moreover, the SUN1-MAJIN interaction was reduced by CDK2 inhibitors.

## Materials and Methods

### Cell Culture and Transfection

HEK293T and Cos7 cells were cultured in Dulbecco’s modified Eagle’s medium (DMEM, Invitrogen, Carlsbad, CA, United States) supplemented with 10% fetal bovine serum (FBS, Invitrogen) and antibiotics (100 U/mL penicillin and 100 μg/mL streptomycin; Invitrogen). Cells were cultured at 37°C with 5% CO_2_ and transfected by the Lipofectamine 2000 reagent (Invitrogen) according to the manufacturer’s instructions. To inhibit the kinase activity of CDK2, 24 h after transfection, cells were treated with roscovitine (50 μM, Selleck, Shanghai, China) or milciclib (50 μM, Selleck) for 4 h. Dimethyl sulfoxide (DMSO) was used as a control.

### Plasmid Construction

Total RNA of the mouse testis was extracted using TRIzol Reagent (Invitrogen), and cDNA was generated using a PrimeScript 1st Strand cDNA Synthesis Kit (TaKaRa, Dalian, China). Full-length mice Sun1 (NM_024451), Terb1 (NM_180958), Terb2 (NM_028914), Majin (NM_001165919), Spdya (NM_029254), Cdk2 (NM_183417), Cyclin E1 (NM_007633) and E2 (NM_001037134) were amplified by PrimeSTAR Max DNA Polymerase (TaKaRa) from cDNA using primers containing specific restriction sites. The genes were cloned into expression plasmids p3 × FLAG-*myc*-CMV-24 (Sigma, St. Louis, MO, United States), pEGFP-C1 (Clontech, Mountain View, CA, United States), pmCherry-C1 (Clontech), and pCMV-N-Myc (Beyotime Biotechnology, Shanghai, China). Truncated mutants were generated by PCR, and deletion mutants were generated using overlapped primers with mutations. The primers were produced by GENEWIZ (Suzhou, China). All plasmids were verified by Sanger sequencing (GENEWIZ).

### Immunoprecipitation Assay

Harvested HEK293T cells were lysed in TNE buffer (10 mM Tris-HCl, pH 7.5, 150 mM NaCl, 1 mM EDTA, and 1% Nonidet P-40) with a protease inhibitor cocktail (Roche, Mannheim, Germany), 1 mM phenylmethylsulfonyl fluoride (PMSF), and 5 mM sodium orthovanadate. The lysates were centrifuged at 18,000 × *g* for 10 min at 4°C, and the supernatants were immunoprecipitated by GFP-Trap (ChromoTek, Munich, Germany) or Anti-FLAG M2 Affinity Gel (Sigma) for 1 h at 4°C on a rotating wheel. The immunoprecipitates were separated by SDS-PAGE and transferred onto nitrocellulose membranes (Sangon Biotech, Shanghai, China).

### Western Blotting

Protein samples were separated on SDS-PAGE gels and then electroblotted onto nitrocellulose membranes. The nitrocellulose membranes were blocked for 1 h in 5% non-fat milk in TBST (10 mM Tris, pH 7.5, 200 mM NaCl, and 0.2% Tween 20) followed by incubation with primary antibodies: mouse anti-GFP antibody (Cat. 66002-1-Ig, Proteintech, Rosemont, IL, United States), rabbit anti-GFP antibody (Cat. ab290, abcam, Cambridge, United Kingdom), mouse anti-FLAG antibody (Cat. F3165, Sigma), rabbit anti-FLAG antibody (Cat. 20543-1-AP, Proteintech), rabbit anti-Myc antibody (Cat. 16286-1-AP, Proteintech), mouse anti-β-Actin antibody (Cat. 66009-1-Ig, Proteintech), rabbit anti-CDK2 antibody (Cat. 10122-1-AP, Proteintech), or rabbit anti-pCDK2 antibody (Cat. ab194868, abcam). Two secondary antibodies were used: goat anti-mouse antibody (Cat. ab216776, IRDye 680RD, abcam) and goat anti-rabbit antibody (Cat. ab216773, IRDye 800RD, abcam). Signals were captured by an Amersham Typhoon (GE Healthcare Bio-Sciences AB, Uppsala, Sweden) and analyzed by ImageQuant TL (GE Healthcare).

### Immunofluorescence

Cos7 cells were cultured on the glass slides. At 24 h after transfection, the cells were fixed in 4% paraformaldehyde (PFA), permeabilized in 0.1% Triton X-100, blocked in 5% BSA and incubated with rabbit anti-FLAG antibody (Proteintech) 4°C overnight, followed by incubation with a cyanine5-conjugated goat anti-rabbit secondary antibody (Cat. A10523, Invitrogen). Nuclei were stained with Hoechst 33342 (Invitrogen). Images were captured by an Axio Imager under an M2 microscope (Carl Zeiss, Göttingen, Germany).

## Results

### SUN1 Is Associated With MAJIN

**FIGURE 1:**
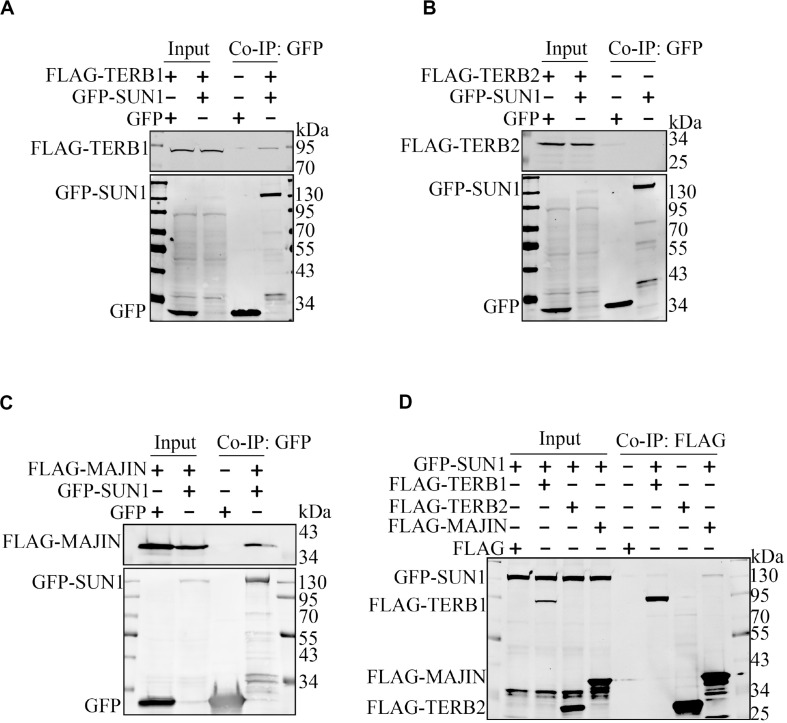
SUN1 is associated with MAJIN. The indicated expression plasmids were transfected into HEK293T cells, and immunoprecipitation was performed to analyze the interactions of SUN1 with TERB1 **(A)**, TERB2 **(B)**, and MAJIN **(C)** by GFP-Trap or Anti-FLAG M2 Affinity Gel **(D)**.

It has been reported that SUN1 interacts with TERB1 (Shibuya et al., 2014), and whether SUN1 interacts with TERB2 or MAJIN is unknown. Immunoprecipitation was performed to examine interactions between SUN1 and the TERB1-TERB2-MAJIN (TTM) complex. The expression plasmid of GFP-SUN1 was co-transfected with FLAG-TERB1, FLAG-TERB2, and FLAG-MAJIN into HEK293T cells, and immunoprecipitated by GFP antibody (Figures 1A–C) and FLAG antibody (Figure 1D), respectively. The results showed that SUN1 had a weak interaction with TERB1 but no interaction with TERB2 (Figure 1). Surprisingly, a strong interaction was found between SUN1 and MAJIN (Figures 1C,D).

### NE Localization of TERB1 and TERB2 Is Dependent on MAJIN but Not SUN1

It has been reported that MAJIN localized to the NE and MAJIN mediated the NE localization of TERB1 and TERB2 (Shibuya et al., 2015), we confirmed these data in Cos7 cells. Expression plasmids of mCherry-tagged TERB1, TERB2, and MAJIN were transfected into the cells. Immunofluorescence show that TERB1 was highly expressed in the nucleus (Supplementary Figure 1Ai), TERB2 was expressed in the whole cell (Supplementary Figure 1Aii), and MAJIN had ring-like localization (Supplementary Figure 1Aiii). Then, expression plasmids of mCherry-MAJIN, FLAG-TERB1, and GFP-TERB2 were transfected into Cos7 cells. The results showed that when TERB1 and TERB2 were co-expressed, no NE localization was detected (Supplementary Figure 1Bi). When TERB1 or TERB2 were co-expressed with MAJIN, TERB2, but not TERB1, was tethered to NE (Supplementary Figures 1Bii,iii). But TERB1 was tethered to NE when co-expressed with MAJIN and TERB2 (Supplementary Figure 1Biv) because MAJIN was associated with TERB2, and TERB2 was associated with TERB1 (Supplementary Figures 1C,D).

SUN1 interacted with TTM complex, and therefore SUN1 may tether the TTM complex to the NE. To test this hypothesis, mCherry-tagged TERB1, TERB2, and MAJIN were co-transfected with GFP-SUN1. The results showed no NE localization of TERB1 or TERB2 when co-expressed with SUN1 (Supplementary Figures 1Ei,ii), but MAJIN showed co-localization with SUN1 (Supplementary Figure 1Eiii). These data suggested that the interaction between SUN1 and TERB1 was not enough to support the NE localization of TERB1.

### Binding Sites Between SUN1 and MAJIN

**FIGURE 2:**
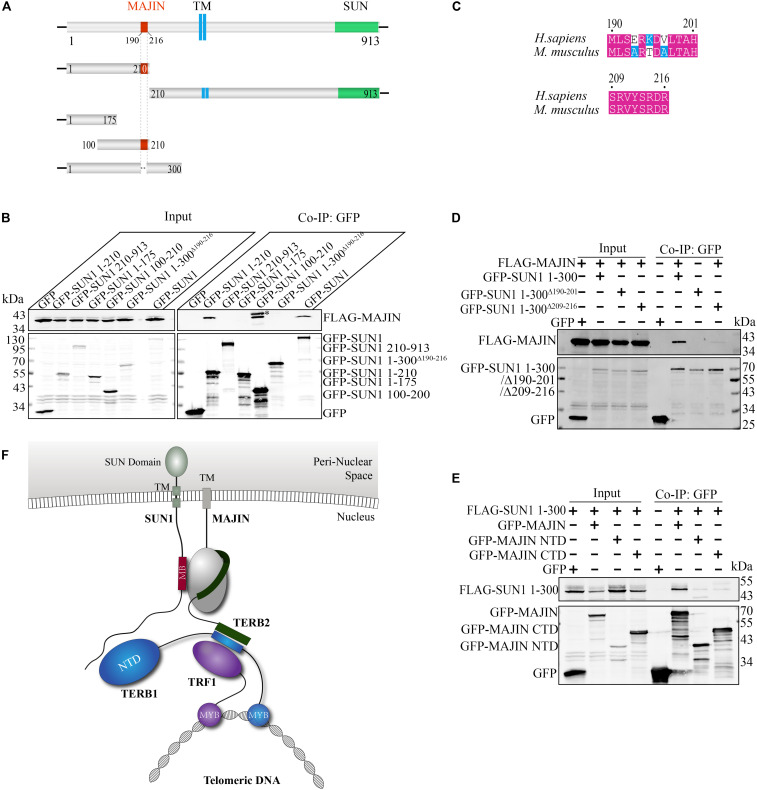
Binding sites between SUN1 and MAJIN. **(A)** Illustration of the MAJIN-binding site at SUN1. The MAJIN-binding site is shown in red, the trans-membrane region is blue, and the SUN domain is green. TM: trans-membrane region. **(B)** The 190–216 Aa of SUN1 is the binding site of MAJIN. ^∗^Indicating the band of GFP-SUN1 100–210 Aa. **(C)** Illustration of the two conserved sequences of SUN1 in the MAJIN binding site. **(D)** The SUN1-MAJIN interaction is affected by deletion of 190–201 and 209–216 Aa. **(E)** Both the NTD and CTD of MAJIN interact with SUN1. **(F)** Illustration of the interaction of SUN1 with MAJIN. The MAJIN-binding site is shown in red and named “MB.”

We mapped the MAJIN-binding site by a panel of mutants of GFP-tagged SUN1 (Figures 2A,B). We found that the N-terminal domain of SUN1 (1–210 amino acid; Aa) interacted with MAJIN. Next, we narrowed down the binding site and found that 1–175 Aa of SUN1 had no interaction with MAJIN, indicating that 175–210 Aa of SUN1 was the binding site of MAJIN (Figure 2B). We found that 190–216 Aa of mouse SUN1 was conserved with human SUN1 (Figure 2C and Supplementary Figure 2). GFP-SUN1 1–300 Aa^Δ 190–216^ with deletion of 190–216 Aa was used to precipitate MAJIN, and we found that this mutant did not interact with MAJIN (Figure 2B). In 190–216 Aa of SUN1, there were two conserved sequences, 190–201 and 209–216 Aa (Figure 2C). GFP-SUN1 1–300 Aa^Δ 190–201^ had no interaction with MAJIN, and GFP-SUN1 1–300 Aa^Δ 209–216^ had very weak interaction (Figure 2D), indicating that 190–216 Aa of SUN1 was the MAJIN-binding site.

We also mapped the SUN1-binding site at MAJIN. We found that both the N-terminal domain (1–100 Aa) and C-terminal domain (100–256 Aa) of MAJIN interacted with SUN1 (Figure 2E).

These results indicated that 190–216 Aa of SUN1 was the MAJIN-binding site, and MAJIN recruited TERB2 and TERB1 to the NE. The interaction between SUN1 and TTM complex is illustrated in Figure 2F.

### SUN1 Interacts With SPDYA

It has been reported that some other proteins are also important for the attachment of telomeres to the NE, such as SPDYA, CDK2, and Cyclin E (Mendez, 2003; Viera et al., 2015; Manterola et al., 2016; Tu et al., 2017). Whether SPDYA, CDK2, or Cyclin E have functions in the connection of the TTM complex with SUN1 is unknown.

**FIGURE 3:**
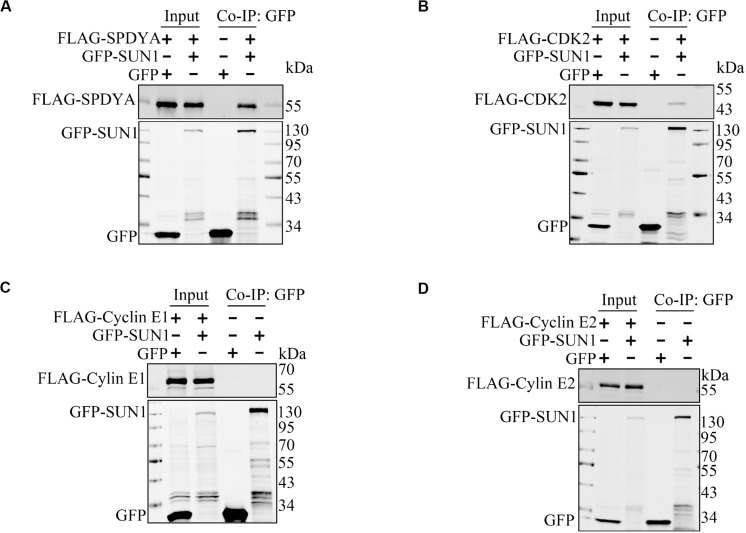
SUN1 interacts with SPDYA. The expression plasmids were transfected into HEK293T cells, and immunoprecipitation was performed to analyze the interaction of SUN1 with SPDYA **(A)**, CDK2 **(B)**, Cyclin E1 **(C)**, and Cyclin E2 **(D)**.

**FIGURE 4:**
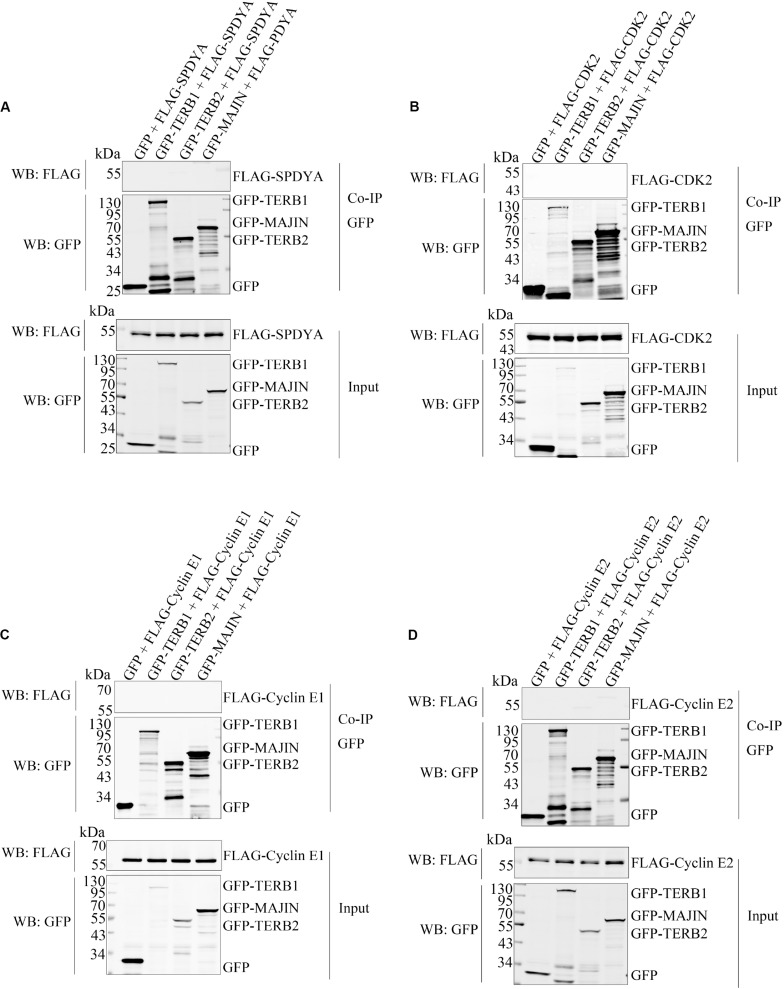
SPDYA, CDK2, and Cyclin E are not associated with TTM complex. The expression plasmids of GFP, GFP-TERB1, GFP-TERB2, and GFP-MAJIN were co-transfected with FLAG-SPDYA **(A)**, FLAG-CDK2 **(B)**, FLAG-Cyclin E1 **(C)**, or FLAG-Cyclin E2 **(D)** into HEK293T cells. Cells were harvested and lysed, 10% cell lysates were used as input, and the remained lysates were incubated with GFP-Trap. After incubation, the GFP-Trap was washed to remove unbound proteins. Then samples of input and Co-IP were subjected to western blotting.

Therefore, we first examined interactions of SPDYA, CDK2, and Cyclin E with SUN1. Immunoprecipitation showed that SPDYA interacted with SUN1 (Figure 3A). CDK2 was associated with SUN1, which was reported in our previous study (Figure 3B). Cyclin E had no interaction with SUN1 (Figures 3C,D). Next, we investigated the interactions of SPDYA, CDK2, and Cyclin E with the TTM complex. Immunoprecipitation showed that SPDYA, CDK2, or Cyclin E did not interact with the TTM complex (Figures 4A–D).

**FIGURE 5:**
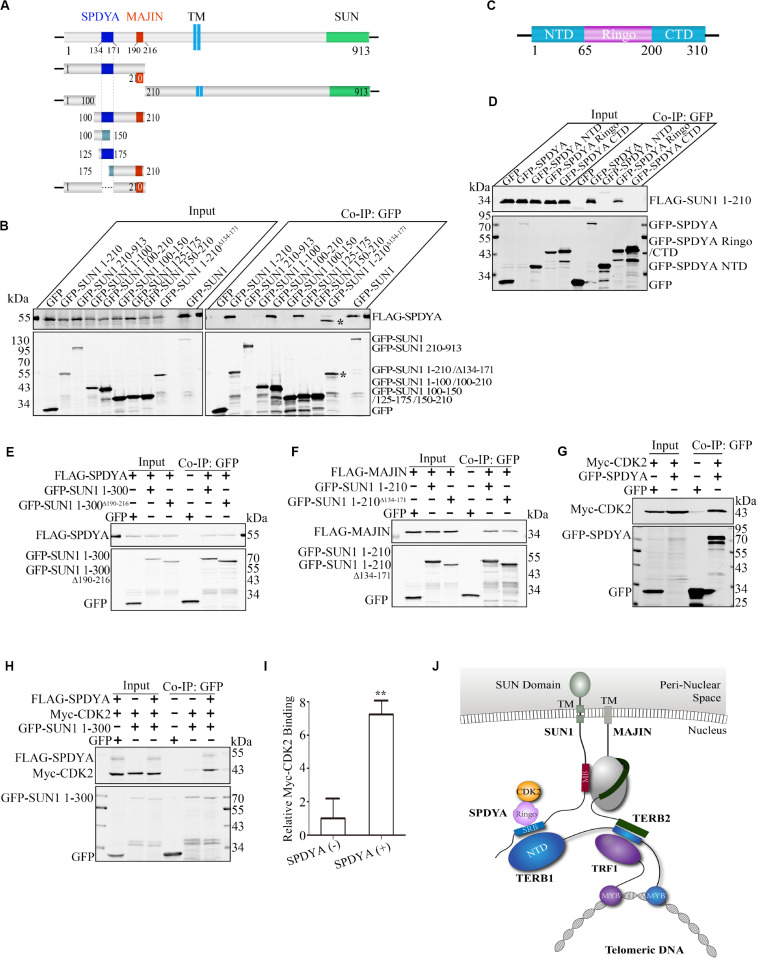
SPDYA recruits CDK2 to SUN1. **(A)** Illustration of the SPDYA-binding site at SUN1. The SPDYA-binding site is shown in dark blue. TM: trans-membrane region. **(B)** The 134–171 Aa of SUN1 in the SPDYA binding site. *Indicating the band of GFP-SUN1 1–210 Aa^Δ 134– 171^. **(C)** Illustration of the SPDYA structure. **(D)** The Ringo domain of SPDYA is the binding site of SUN1. **(E)** The interaction of SUN1 mutant with MAJIN-binding site deletion between SPDYA. **(F)** The interaction of SUN1 mutant with SPDYA-binding site deletion between MAJIN. **(G,H)** SPDYA interacts with CDK2 and recruits CDK2 to SUN1. **(I)** The histogram shows Myc-CDK2 binding in **(H)**, data are presented as mean ± SEM with three repeats. **P < 0.01. **(J)** Illustration of the interaction of SUN1 with MAJIN and SPDYA-CDK2. The SPDYA Ringo domain binding site of SUN1 is shown in blue and name “SRB.”

We mapped the SPDYA-binding site as described previously (Figure 2). The results showed that SPDYA bound to 125–175 Aa at the N-terminal of SUN1 (Figures 5A,B and Supplementary Figure 3). We found that 134–171 Aa of mouse SUN1 was conserved with human SUN1 (Supplementary Figure 2). GFP-SUN1 1–210 Aa^Δ 134–171^ with deletion of 134–171 Aa was used to precipitate SPDYA, and the mutant had no interaction with SUN1, indicating that 134–171 Aa of SUN1 was the SPDYA-binding site (Figure 5B). Next, the SUN1-binding site of SPDYA was mapped. The results showed that the Ringo domain of SPDYA was the SUN1-binding site (Figures 5C,D).

We found that both MAJIN and SPDYA bound to the N-terminal domain of SUN1 and the two binding sites were close to each other (Figures 2, 5A,B). Therefore, we examined the interactions of SUN1 with SPDYA and MAJIN using SUN1 mutants with MAJIN or SPDYA binding site deletion. The results showed that the interaction of SUN1 between SPDYA was not affected by the deletion of MAJIN-binding site (Figure 5E), and the interaction of SUN1 between MAJIN was slightly decreased by the deletion of SPDYA-binding site (Figure 5F).

SPDYA recruits and activates CDK2 (Tu et al., 2017), indicating that SPDYA may enhance the interaction of SUN1 with CDK2. Immunoprecipitation showed that SPDYA interacted CDK2 (Figure 5G), and SPDYA promoted the interaction of SUN1 with CDK2 (Figures 5H,I).

These results indicate that 134–171 Aa of SUN1 is associated with the Ringo domain of SPDYA, and SPDYA recruits CDK2 to SUN1. The interaction between SUN1, SPDYA-CDK2, and the TTM complex is illustrated in Figure 5J.

### The Interaction of SUN1 With MAJIN Is Reduced by CDK2 Inhibitors

**FIGURE 6:**
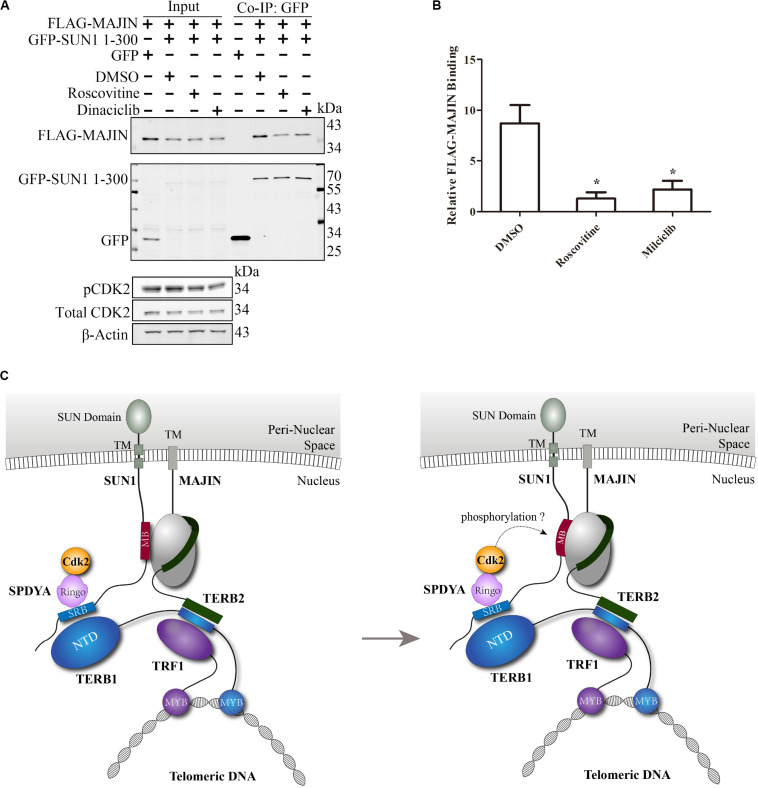
The interaction of SUN1 with MAJIN is reduced by CDK2 inhibitors. **(A)** The SUN1-MAJIN interaction is decreased by CDK2 inhibitors. **(B)** The histogram shows FLAG-MAJIN binding in **(A)**, data are presented as mean ± SEM with three repeats. *P < 0.05. **(C)** Illustration of the interaction of SUN1 with MAJIN regulated by SPDYA-CDK2.

SPDYA recruits CDK2 to SUN1, and the MAJIN-binding site is adjacent to the SPDYA-binding site, suggesting that the interaction of SUN1 with MAJIN may be regulated by CDK2, especially because the connection of telomeres with the NE is dependent on CDK2 and SPDYA. GFP-SUN1 1–300 and FLAG-MAJIN were co-transfected, followed by treatment with roscovitine and milciclib, two inhibitors of CDK2 and then immunoprecipitation assay was carried out. The results showed that the interaction of SUN1 with MAJIN was decreased by the inhibition of CDK2 activity (Figures 6A,B). However, the CDK2 phosphorylation sites of SUN1 and MAJIN are unknown. These results indicate that the interaction of SUN1 with MAJIN is regulated by SPDYA-CDK2 (Figure 6C).

## Discussion

During meiosis, telomeres are tethered to the NE. This tethering is critical for meiotic chromosome alignment and synaptic pairing of the homologs (Harper et al., 2004; Lee et al., 2015; Zeng et al., 2017). Many proteins are involved in this tethering process, such as the LINC (linker of nucleoskeleton and cytoskeleton) complex, the “adaptor,” and the shelterin complex (Shibuya et al., 2015; Burke, 2018; Li and Liu, 2020). The LINC complex consists of Klarsicht, Anc-A, and Syne Homology (KASH) and Sad1 and UNC-84 (SUN) domain proteins. The KASH domain proteins are associated with the outer nuclear membrane, and the SUN domain proteins are in the inner nuclear membrane. The LINC complex provides coupling between the cytoskeleton and nucleoskeleton. During meiosis in the mouse, the LINC complex consists of KASH5 and SUN1 or SUN2, and the SUN domain proteins are the anchoring site of telomeres at the NE (Ding et al., 2007; Wang et al., 2012; Horn et al., 2013; Link et al., 2014). The “adaptor” consists of TERB1, TERB2, and MAJIN (da Cruz et al., 2020). The shelterin complex has six proteins, containing TRF1, TRF2, POT1, RAP1, TIN2, and TPP1. The shelterin complex is localized to the chromosome end and protects telomeric DNA (Palm and de Lange, 2008). The TTM complex acts as a “linker” and bridges the LINC and shelterin complexes together. The connection between TTM and shelterin complexes is well-known, which is mediated by TERB1 and TRF1 (Shibuya et al., 2014; Long et al., 2017; Pendlebury et al., 2017; Zhang et al., 2017; Dunce et al., 2018). However, the connection between the TTM and LINC complex was not fully understood.

In this study, we examined the association between SUN1 and TTM complex by immunoprecipitation assay. We found that SUN1 was associated with TERB1 (Figures 1A,D), as reported previously (Shibuya et al., 2014). Moreover, SUN1 was associated with MAJIN, and the SUN1-MAJIN interaction was stronger than SUN1-TERB1 (Figures 1A,C,D), but SUN1 was not associated with TERB2 (Figure 1B). It has been reported that SUN2 connects a portion of telomeres to the NE in *Sun1* knockout mice (Link et al., 2014), suggesting that SUN2 also mediates the telomere-NE connection. In fact, we found that SUN2 was associated with the TTM complex, and how SUN2 mediates the telomere-NE connection is under study (unpublished data).

The SUN1-MAJIN interaction may be more important than the SUN1-TERB1 interaction in the bridge of LINC and shelterin complexes, because the interaction of SUN1-MAJIN was stronger than TERB1 (Figure 1). Moreover, MAJIN has a trans-membrane region and is co-localized with SUN1 to the NE, but TERB1 and TERB2 are not co-localized with SUN1 (Supplementary Figure 1), indicating that MAJIN plays a major role in the association of the SUN1-TTM complex. Additionally, in the *Terb1* knockout mouse, overexpressed GFP-MAJIN in spermatocytes is pulled to the centrosome pole and exhibits a crescent-shape distribution but not in the *Sun1* knockout mouse (Shibuya et al., 2015), suggesting that MAJIN interacts directly with SUN1, not mediated by TERB1 as reported previously.

CDK2 is a cyclin-dependent kinase that is required for the telomere-NE connection (Ortega et al., 2003; Viera et al., 2009, 2015; Palmer et al., 2019). SPDYA and Cyclin E are activators of CDK2 and also critical for the telomere-NE connection (Martinerie et al., 2014; Manterola et al., 2016; Mikolcevic et al., 2016; Tu et al., 2017). However, the mechanisms of CDK2 and its activators in promotion of the telomere-NE connection are unclear. We found that SPDYA interacted with SUN1 (Figure 3A), and CDK2 was associated with SUN1 (Figure 3B), as we reported previously (Liu et al., 2014). However, Cyclin E did not interact with SUN1 (Figures 3C,D). SPDYA, CDK2, or Cyclin E did not interact with the TTM complex (Figures 4A–D). In *Cyclin E* knockout mice, the telomere localization of CDK2 is reduced (Martinerie et al., 2014), and the shelterin complex protein, TRF2 and RAP1, are decreased (Manterola et al., 2016), suggesting that Cyclin E-CDK2 mainly regulates the connection of TTM with shelterin complexes. Moreover, in the *Spdya* knockout mouse, TERB1 and MAJIN are still localized at the telomere, but not SUN1 (Tu et al., 2017). These results indicate that the connection between SUN1 and TTM complexes may be promoted by SPDYA-CDK2 but not Cyclin E-CDK2.

We mapped the binding site and found that 190–216 and 134–171 Aa of SUN1, which are conserved between mouse and human (Supplementary Figure 2), were the binding sites of MAJIN and SPDYA, respectively (Figures 2, 5). Interestingly, the Ringo domain of SPDYA interacted with SUN1 (Figure 5D). The Ringo domain is required for telomere localization of SPDYA (Tu et al., 2017). These results indicate that the 134–171 Aa of SUN1 is the anchoring site of SPDYA at the NE and may be responsible for the telomere localization of SPDYA. It has been reported that SPDYA interacts with TRF1 (Wang et al., 2018), suggesting that this interaction is also responsible for telomere localization of SPDYA; however, whether the Ringo domain participates in the interaction of SPDYA-TRF1 is still unknown.

It has been reported that SPDYA recruits CDK2 to telomeres (Tu et al., 2017), implying that SPDYA promotes the interaction of SUN1 with CDK2. To test this hypothesis, the interaction of SPDYA with CDK2 was confirmed by immunoprecipitation (Figure 5G). Next, we found that SPDYA increased the interaction of SUN1 with CDK2 (Figures 5H,I), suggesting that SPDYA interacts with 134–171 Aa of SUN1 via the Ringo domain, and then SPDYA recruits CDK2 to SUN1 to localize to the telomere.

The kinase activity of CDK2 is important for meiosis and spermatogenesis (Liu et al., 2014; Wang et al., 2014; Chauhan et al., 2016; Palmer et al., 2019). It has been reported that CDK2 phosphorylates SUN1 (Viera et al., 2015; Mikolcevic et al., 2016), but the function of SUN1 phosphorylation by CDK2 is unknown. In this study, we found that the interaction of SUN1 with MAJIN is reduced by the CDK2 inhibitors (Figures 6A,B), implying that SPDYA-CDK2 may promote the telomere-NE connection by regulating the SUN1-MAJIN interaction (Figure 6C).

In summary, we revealed the mechanism of telomere tethering to the NE during meiosis, which is mediated by the interaction between SUN1 and MAJIN. SPDYA binds to SUN1 via the Ringo domain and recruits CDK2 to the telomere to tether telomeres to the NE.

## Data Availability Statement

All datasets generated for this study are included in the article/Supplementary Material.

## Author Contributions

GW and FS conceived and designed the study. GW and XW performed immunoprecipitation and immunofluorescence assay and wrote the manuscript. LZ, SG, DY, and AL performed cell culture and transfection, and plasmid construction. FS revised the manuscript and approved the final version. All authors reviewed the final version of the manuscript.

## Conflict of Interest

The authors declare that the research was conducted in the absence of any commercial or financial relationships that could be construed as a potential conflict of interest.

## Supplementary Material

FIGURE S1 Nuclear envelope localization of TERB1 and TERB2 is dependent on MAJIN but not SUN1. **(A,B,E)** Expression plasmids were transfected into Cos7 cells, and then immunofluorescence assays were carried out. Nuclei were stained with Hoechst 33342. mCh: mCherry. Scale bar = 20 μm. **(A)** Cellular localization of TERB1, TERB2, and MAJIN. **(B)** Nuclear envelope localization of TERB1 and TERB2 is dependent on MAJIN. **(E)** Nuclear envelope localization of TERB1 and TERB2 is independent of SUN1. **(C,D)** Expression plasmids were transfected into HEK293T cells, and immunoprecipitation was performed to analyze the interactions of TERB1-TERB2-MAJIN.

FIGURE S2 Sequence alignment of human and mouse SUN1.

FIGURE S3 Uncropped images presented in Figure 4B. ^∗^Indicating the band of GFP-SUN1 1–210 Aa^Δ134–171^.
