# Supplementary figures and images for: Tethering of Telomeres to the Nuclear Envelope Is Mediated by SUN1-MAJIN and Possibly Promoted by SPDYA-CDK2 During Meiosis
Source: Front Cell Dev Biol. 2020 Sep 4;8:845. doi: 10.3389/fcell.2020.00845 (PMC7509418; doi:10.3389/fcell.2020.00845)

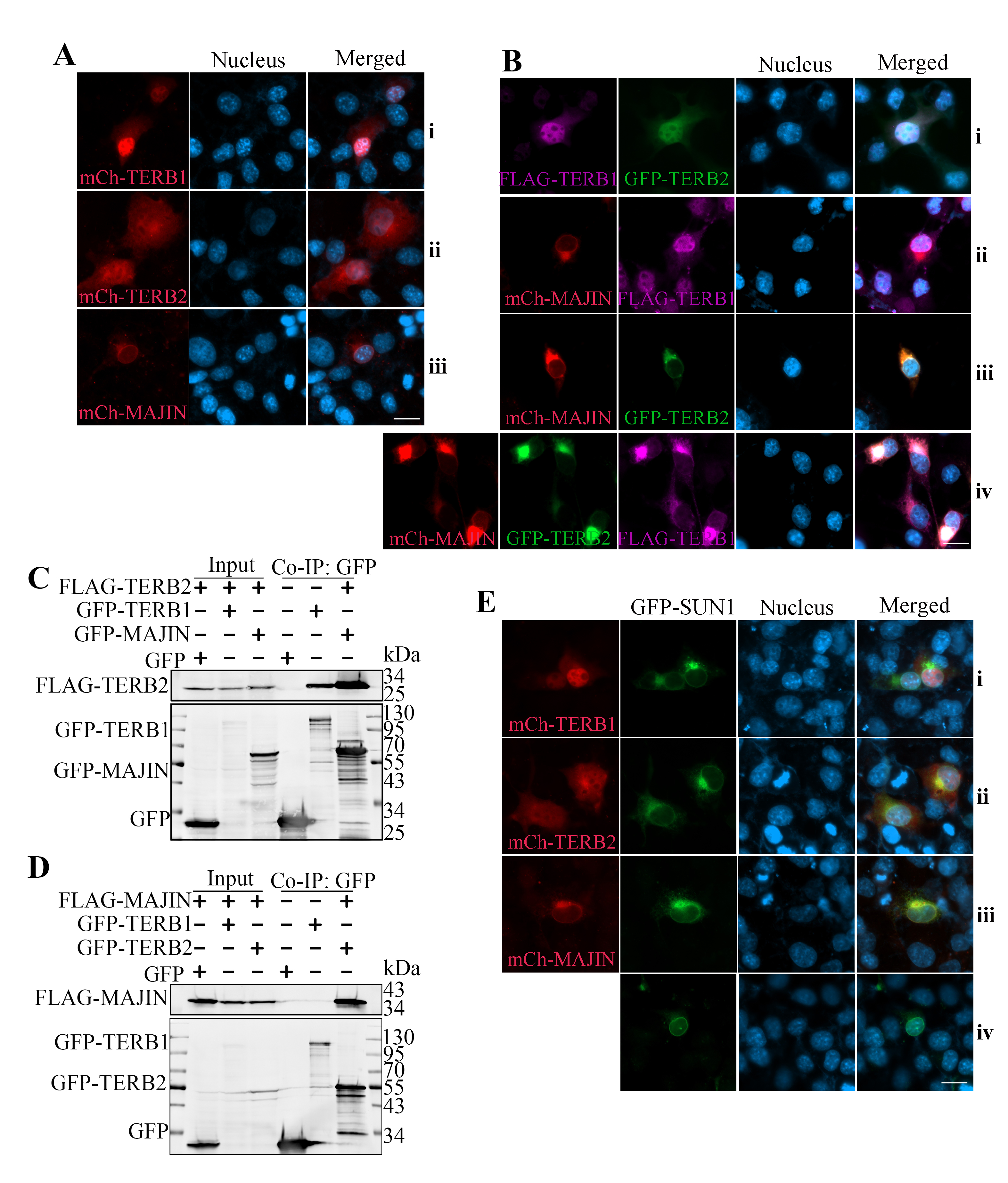

Supplement: FIGURE S1 — Nuclear envelope localization of TERB1 and TERB2 is dependent on MAJIN but not SUN1. (A,B,E) Expression plasmids were transfected into Cos7 cells, and then immunofluorescence assays were carried out. Nuclei were stained with Hoechst 33342. mCh: mCherry. Scale bar = 20 μm. (A) Cellular localization of TERB1, TERB2, and MAJIN. (B) Nuclear envelope localization of TERB1 and TERB2 is dependent on MAJIN. (E) Nuclear envelope localization of TERB1 and TERB2 is independent of SUN1. (C,D) Expression plasmids were transfected into HEK293T cells, and immunoprecipitation was performed to analyze the interactions of TERB1-TERB2-MAJIN. [file Image_1.JPEG]

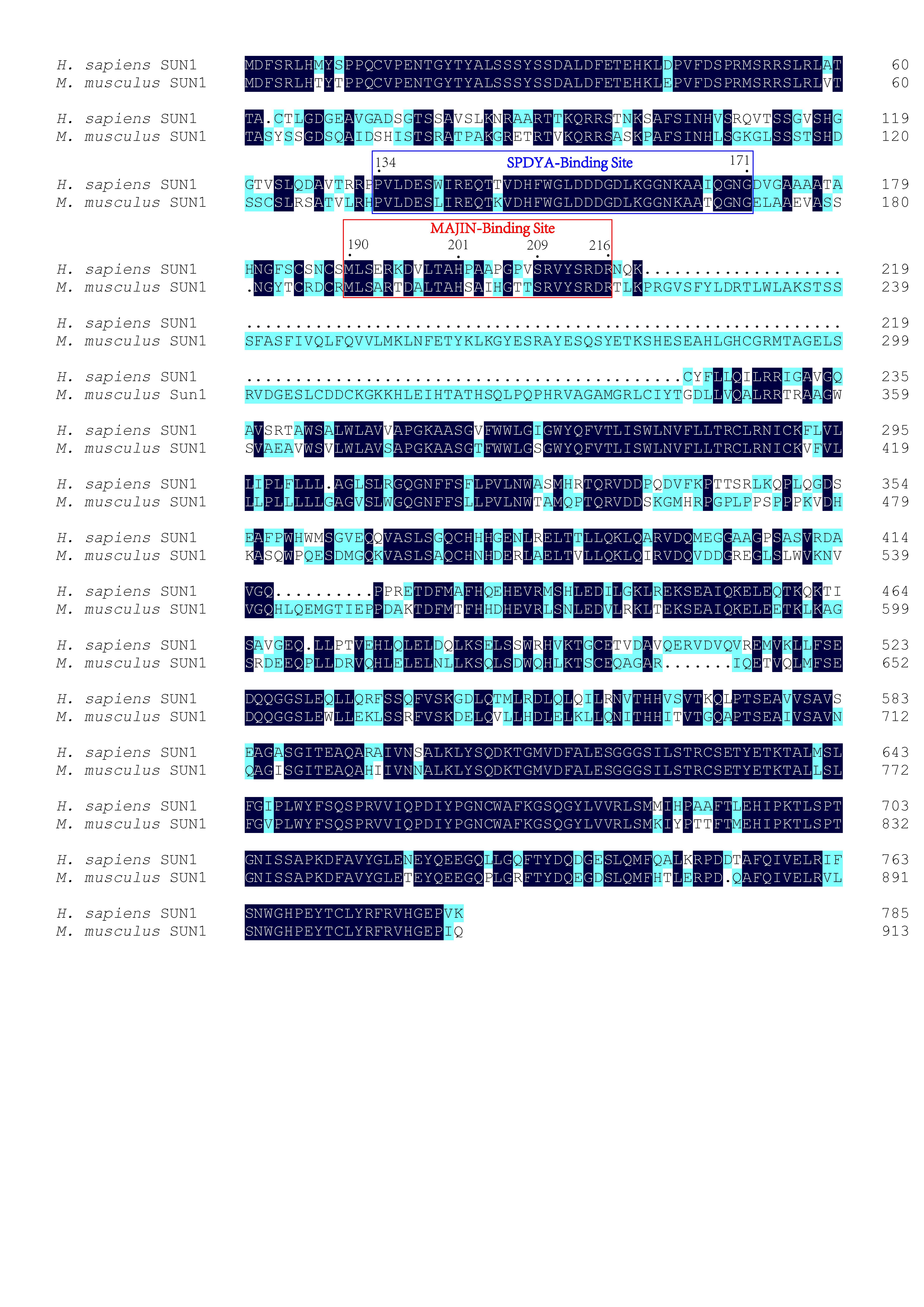

Supplement: FIGURE S2 — Sequence alignment of human and mouse SUN1. [file Image_2.JPEG]

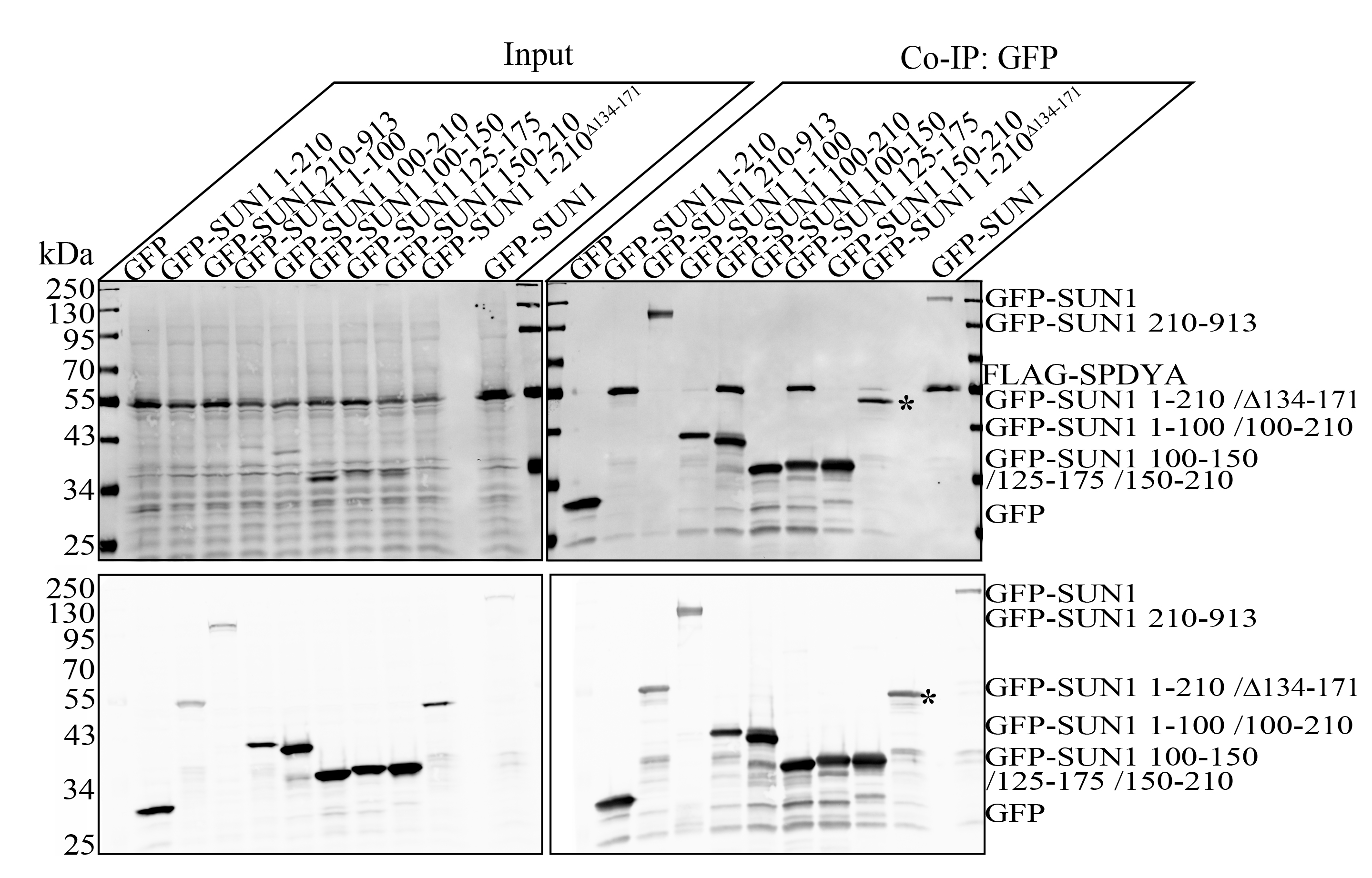

Supplement: FIGURE S3 — Uncropped images presented in Figure 4B. ∗Indicating the band of GFP-SUN1 1–210 AaΔ134–171. [file Image_3.JPEG]
